# Estimation of Electrostatic Interaction Energies on a Trapped-Ion Quantum Computer

**DOI:** 10.1021/acscentsci.4c00058

**Published:** 2024-03-26

**Authors:** Pauline J. Ollitrault, Matthias Loipersberger, Robert M. Parrish, Alexander Erhard, Christine Maier, Christian Sommer, Juris Ulmanis, Thomas Monz, Christian Gogolin, Christofer S. Tautermann, Gian-Luca R. Anselmetti, Matthias Degroote, Nikolaj Moll, Raffaele Santagati, Michael Streif

**Affiliations:** † QC Ware Corp., Palo Alto, California 94306, United States; ‡ QC Ware Corp., Paris 75003, France; § Alpine Quantum Technologies GmbH, 6020 Innsbruck, Austria; ∥ Covestro Deutschland AG, 51373 Leverkusen, Germany; ⊥ Medicinal Chemistry, Boehringer Ingelheim Pharma GmbH & Co. KG, 88397 Biberach, Germany; # Quantum Lab, Boehringer Ingelheim, 55218 Ingelheim am Rhein, Germany; △ Institut für Experimentalphysik, Universität Innsbruck, 6020 Innsbruck, Austria; ▲ Department of General, Inorganic and Theoretical Chemistry, University of Innsbruck, 6020 Innsbruck, Austria

## Abstract

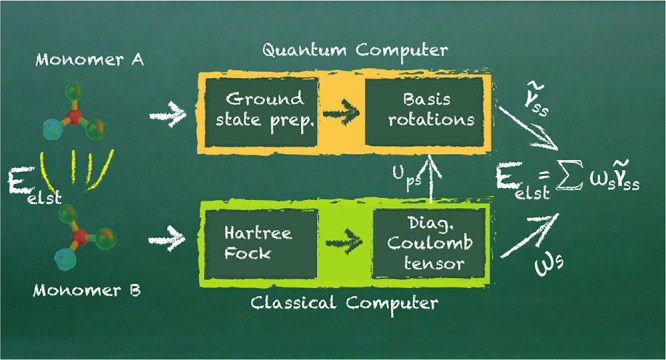

We present the first hardware implementation of electrostatic interaction energies by using a trapped-ion quantum computer. As test system for our computation, we focus on the reduction of NO to N_2_O catalyzed by a nitric oxide reductase (NOR). The quantum computer is used to generate an approximate ground state within the NOR active space. To efficiently measure the necessary one-particle density matrices, we incorporate fermionic basis rotations into the quantum circuit without extending the circuit length, laying the groundwork for further efficient measurement routines using factorizations. Measurements in the computational basis are then used as inputs for computing the electrostatic interaction energies on a classical computer. Our experimental results strongly agree with classical noise-less simulations of the same circuits, finding electrostatic interaction energies within chemical accuracy despite hardware noise. This work shows that algorithms tailored to specific observables of interest, such as interaction energies, may require significantly fewer quantum resources than individual ground state energies would require in the straightforward supermolecular approach.

## Abstract 2

The first demonstration of the computation of electrostatic interaction energies on a trapped-ion quantum computer, tested on the reduction of NO to N_2_O, shows strong agreement with classical simulations.

## 1 Introduction

The potential of quantum computers to simulate molecules is promising for many applications in industry and research.^1−3^ While quantum computers that could simulate classically intractable molecules have yet to be built, the task of identifying relevant industrial applications of these future machines is equally crucial. This goal involves not only identifying classically challenging molecules that could benefit from quantum computations^4−7^ but also developing quantum algorithms which perform the computations required for specific applications in industry.

To date, most quantum algorithms have been developed to capture more accurate ground state total energies.^8,9^ However, accurate computations of properties beyond ground state energy are essential for many industrial applications.^10−12^ A prime example is the estimation of interaction energies between two molecules, a critical first step in computational drug design for ranking the efficacy of ligands against a target such as a protein.^13^ On a quantum computer, interaction energies could be obtained through three distinct ground state energy computations: one for each molecule and one for the entire system. Given that interaction energies typically constitute only a fraction of the total ground state energies, this method—known as the supermolecular approach—requires a high precision in each ground state energy calculation.^14,15^

Symmetry-adapted perturbation theory (SAPT) offers an alternative approach by expressing the interaction energy from a perturbative treatment of the intermolecular potential.^16−18^ Recent theoretical advancements have laid the foundation for harnessing emerging quantum computing capabilities to compute more accurate interaction energies via SAPT.^19,20^ Specifically, it was demonstrated that a near-term quantum algorithm, the variational quantum eigensolver (VQE),^8^ could enhance the accuracy of SAPT calculations and provide an alternative to the supermolecular approach. However, this was tested only via the emulation of the quantum computer on classical computers.

In this work, we present the first experimental demonstration toward the computation of SAPT energies on a quantum computer, focusing specifically on the quantum computation of the electrostatic energy. We note that the computation of the electrostatic term by itself does not require symmetry adaption. However, we still refer to SAPT interaction energies as this work presents the first step toward the goal of implementing the full second order SAPT energies on quantum hardware.^19,20^

An important part of this quantum algorithm is its embedding into a classical framework. Therefore, it is desirable to apply it to molecules whose sizes and complexities are representative of standard problems in industrial use cases. This allows us to resolve any classical bottleneck, ensuring a smooth transition to an era with improved quantum hardware. Hence, as a test system for our experiments, we focus on the active site of a nitric oxide reductase (NOR), a key player in the chemical reaction that reduces nitric oxide (NO) to nitrous oxide (N_2_O), i.e., an important step of the nitrogen cycle. Several intermediates in the catalytic cycle have electronic structures that are strongly correlated and, thus, challenging to simulate with classical methods. As a result, the NOR active site serves as a valuable benchmark system for quantum algorithms. Notably, NOR is a member of the cytochrome P450 superfamily, which is renowned for its monoxygenases that play a central role in drug metabolism. The fact that NOR belongs to the P450 superfamily renders the exploration of the catalytic cycle of NO reduction not only intrinsically valuable but also broadly significant due to its structural parallels with other P450 enzymes.

In order to make our computations possible on today’s quantum computers with limited qubit counts and gate fidelities, we rely on classical quantum chemistry methods to preprocess and heavily simplify the system. Specifically, while we treated the studied molecules with more than 1000 orbitals classically, we only utilize four orbitals (mapped to eight qubits) to account for the molecule’s strong correlation on the quantum computer. Despite the quantum algorithm modeling only a very small part of the system, a task that could be readily handled by any classical computer, our work serves as the first demonstration of such a computation on quantum hardware. An interesting feature emerging from this experiment is the measurement in a single basis allowed by the implementation of a fermionic basis change in the quantum circuit. The same circuit primitives could also be used to measure a two-particle density matrix (2-PDM) with the help of double factorization,^9,21,22^ greatly reducing the number of measurement circuits compared to the “naïve” approach. This feature also highlights our work as a demonstration of experimental capabilities relevant beyond the SAPT framework.

In section 2, we review the SAPT method and explain how to efficiently compute the first term of its perturbation series, the electrostatic contribution. Next, in section 3, we highlight the crucial classical steps that account for the preparation of the molecular systems and the optimization of quantum circuits. Finally, in section 4, we review the implementation of quantum circuits on quantum hardware and discuss the experimental results.

## 2 Quantum Computations of the Electrostatic Energy

The interaction energy between two monomers A and B is given in the supermolecular approach by
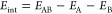
1where *E*_AB_, *E*_A_, and *E*_B_ denote the ground state energies of the dimer, monomer A, and monomer B, respectively. Consequently, using this approach, the computation of the interaction energy requires three separate ground-state energy calculations, one of which is performed on a larger dimer system. An alternative approach to computing interaction energies is SAPT.^16,17^ In SAPT, the interaction between the two molecules is treated as a perturbation, and the solution of the symmetrized Rayleigh–Schrödinger equation yields the interaction energy as a perturbation series, written in *polarization* terms, *E*_pol_ and *exchange* terms, *E*_exch_, as
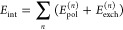
2where *n* is the perturbation order.

The polarization and exchange terms of eq 2 are commonly labeled to their interaction energy kind. The four main interaction types are the electrostatic energy, the exchange energy, the induction energy and the dispersion energy. The exact expression of these main terms depends on the level of truncation in the chosen SAPT method. We direct the reader to refs (23) and (24) for a more detailed introduction to SAPT. It is important to remark here that, on top of providing insight into the underlying forces of the intermolecular interaction, this approach also eliminates the need for a ground state energy calculation of the dimer system and the propagation of large errors from energy calculations into typically small energy differences.

In this study, we focus on the quantum computation of a single interaction type, *E*_elst_, which describes the electrostatic energy between the two monomers. We employ the full configuration interaction (FCI) SAPT formalism of ref (25) wherein a complete (or VQE-type near-complete) treatment of electron correlation is assumed at each level of intermolecular perturbation. In this case, the electrostatic energy can be calculated via
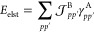
3where γ_*pp*′_^A^ is the spin-summed one-particle density matrix (1-PDM) of monomer A in a (nonorthogonal) spin-restricted spatial “atomic orbital (AO)” basis {ϕ_*p*_(*r⃗*)} and
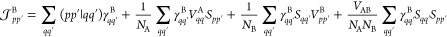
4is the generalized electrostatic potential matrix. Here, *N*_A_ and *N*_B_ are the numbers of electrons of monomer A and monomer B, respectively. The first term comprises the two-body integrals (*pp*′|*qq*′) and the spin-summed 1-PDM of monomer B, γ_*qq*′_^B^. In the second and third terms, *V*_*pp*′_^X^ is the nuclear potential from monomer X, and *S*_*pp*′_ is the overlap matrix in the AO basis. In the last term, *V*_AB_ is the intermonomer nuclear repulsion term. All formal definitions can be found in section S1 of the Supporting Information. In this work, the orbital basis is always defined as the dimer basis.

The expression of eq 3 is particularly advantageous when monomer B is well described by classical quantum chemistry methods such as Hartree–Fock(HF), density functional theory (DFT), or other low polynomial-scaling approaches as, in this case,  can easily be obtained in classical preprocessing. The remaining task is to find the (crucial) interaction of the electronic density of A with the full electrostatic potential of B. To do so, γ_*pp*′_^A^ needs to be computed. When the ground state wave function of A, |Ψ_A_⟩, is represented in whole, or in part, on a quantum computer, the use of an orthonormal “molecular orbital (MO)” spatial basis is commonly required. The MO basis is defined by
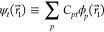
5with the orthonormality condition
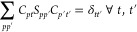
6The 1-PDMs in the AO and MO basis are then related by
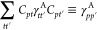
7In the MO basis, eq 3 becomes
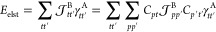
8The rewriting of *E*_elst_ in the MO basis, in eq 8, is interesting because it makes apparent that a different set of MOs, *C̅*_*pv*_, can be chosen such that  is diagonal, i.e.,
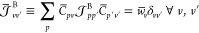
9This “electrostatic potential natural orbital basis” is simply obtained by rotating the canonical MOs as
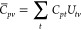
10where *U*_*tv*_ are the eigenvectors of  and *w̅*_*v*_ are the corresponding eigenvalues. Finally, eq 8 becomes
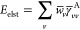
11with γ̅_*vv*_^A^ = ∑_*tt*′_*U*_*tv*_ γ_*tt*′_^A^*U*_*t*′*v*_.

**Figure 1:**
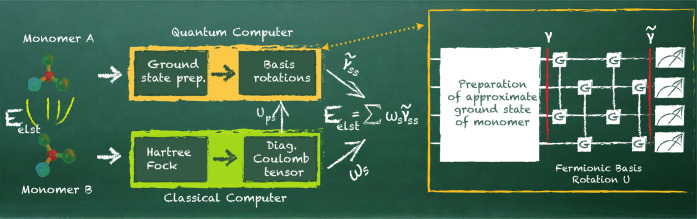
Visualization of the quantum computation of the electrostatic interaction energy. (Left) The output of the classical Hartree–Fock calculations is fed into the quantum computer, where a previously generated approximate ground state of monomer A is rotated in the basis given in eq 11. Measuring the diagonal elements of the 1-RDM allows for an efficient calculation of the electrostatic energy. (Right) Visualization of the quantum circuit, including preparing the approximate ground state and the subsequent fermionic basis rotation; see also Figure 3.

Importantly, when the wave function is represented on a quantum computer with a spin-restricted Jordan-Wigner formalism, the diagonal elements of the MO 1-PDM correspond to commuting, diagonal, single-particle Pauli measurements:
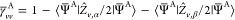
12where the Pauli operator *Ẑ*_*v*,α_ acts on the qubit representing MO *v* with spin α and |Ψ̅^A^⟩ is the ground state of A in the electrostatic potential natural orbital basis. Note that the determination of the ground state |Ψ_A_⟩ can still be performed as usual with the canonical MOs, and the transformation to the electrostatic potential natural orbitals can simply be implemented by decomposing the fermionic basis rotation *U*_*tv*_ into Givens rotations^26,27^ and appending them to the quantum circuit right before the measurements. We provide a visualization of the computation in Figure 1.

Ultimately, we see that this procedures allows the obtaining of *E*_elst_ from a single set of measurements since all the operators required to reconstruct γ_*vv*_^A^, as defined in eq 12, commute with each other. The employed method contrasts with the “naïve” approach where the number of distinct measurement circuits to reconstruct the 1-PDM grows quadratically with the number of orbitals *N*.

In comparison, the supermolecular approach, as per eq 1, requires the calculation of the ground state energies of both the monomer and dimer systems, which confers a clear advantage to the perturbative alternative. Naively, this method requires up to  measurement circuits. However, an interesting parallel with the present work is that the same change of fermionic basis approach can be used to measure the 2-PDM with the help of double factorization,^9,21,22^ reducing the number of measurement circuits. Furthermore, randomizing over these basis rotations would allow for a classical shadow approach only scaling with the number of occupied fermionic modes.^28^ We note that performing the VQE computation in the electrostatic potential natural orbital basis would eliminate the need for applying the basis rotation on the quantum computer. However, this particular basis might not be optimal for preparing approximate ground states of the Hamiltonian. Additionally, for higher-order terms such as the exchange energy, we would require  different bases,^19^ making it impossible to fix a single basis a priori.

## 3 Classical Processing

As a test system, we exploit the reduction of NO to N_2_O catalyzed by NOR. Details on this reaction, such as the proposed catalytic cycle are discussed in section S2 of the Supporting Information. Reference (29) discusses the importance of intermolecular interactions in the reaction mechanism. For instance, water molecules surrounding the NOR active site form a hydrogen bonding network and act as a proton delivery pathway for the formation of one key intermediate. One intermediate step involves a hydride transfer from a surrounding NADH molecule to the NO-bound heme (see section S3 of the Supporting Information). The NO-bound heme is expected to exhibit strong correlations and hence can be a good target for quantum computations. The strong correlation arises as π/π*orbitals of both NO and porphyrin moieties are known to mix with the 3d orbitals of the Fe center.^5,30,31^ In this context, we inquire whether electrostatic interactions favor the stabilization of the NADH molecule in the heme pocket. In practice, this involves calculating the difference in electrostatic energies between both intermediates, denoted as Δ*E*_elst_ = *E*_elst_(B) – *E*_elst_(A).

**Figure 2:**
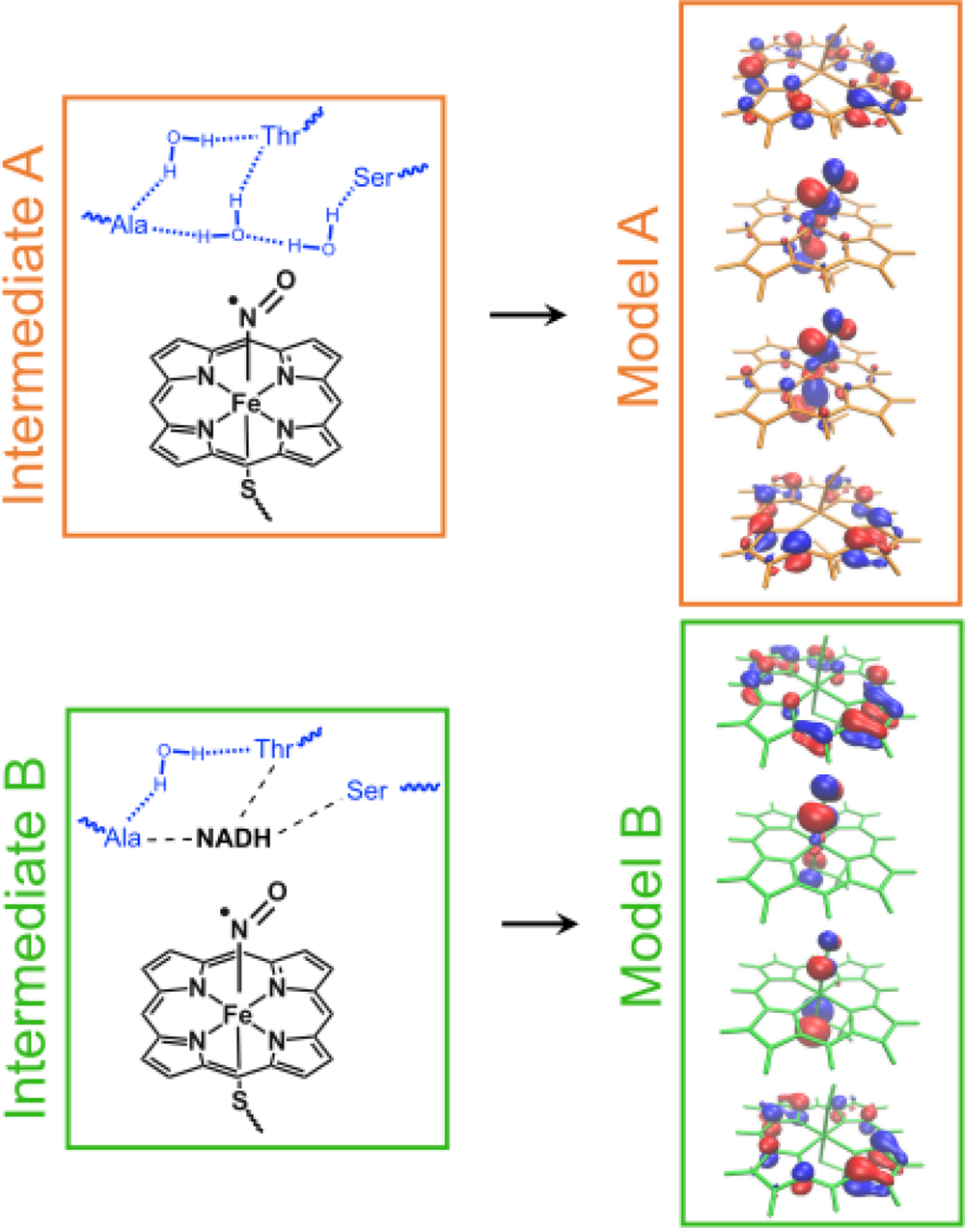
(Left) 2D representations of intermediates A and B. They include the NO-bound heme, three surrounding amino acids, water molecules, and NADH (if present). In both cases, monomers A and B are shown in black and blue, respectively. (Right) The four orbitals in the active space of monomer A for each intermediate.

The preparation of the model systems for the two intermediates of interest, shown in Figure 2, is discussed in great detail in section S2 of the Supporting Information. To calculate the electrostatic interactions, we break down both intermediates into two separate monomers. For intermediate A, the first monomer consists of the NO-bound heme, while the second monomer encompasses the amino acids and water molecules. In the case of intermediate B, the first monomer includes both the NO-bound heme and the NADH molecule, while the amino acids and the remaining water molecule form the second monomer. In both cases, the ground state wave function of the first monomer is partially treated with a quantum computer. To match with the hardware constrains, we select a small four-electrons-in-four-MOs (4e, 4o) active space. Capturing the important electronic correlations in our systems would obviously require a much larger active space,^5^ and the choice we make is strictly driven by the experimental setup. However, the orbitals selected by looking at the natural orbital occupation numbers (see section S2 of the Supporting Information and Figure 2) include character from the iron 3d, NO (anti)bonding π, porphyrin (anti)bonding π orbitals which govern the strong corrleation in this system.^5,30,31^

**Figure 3:**
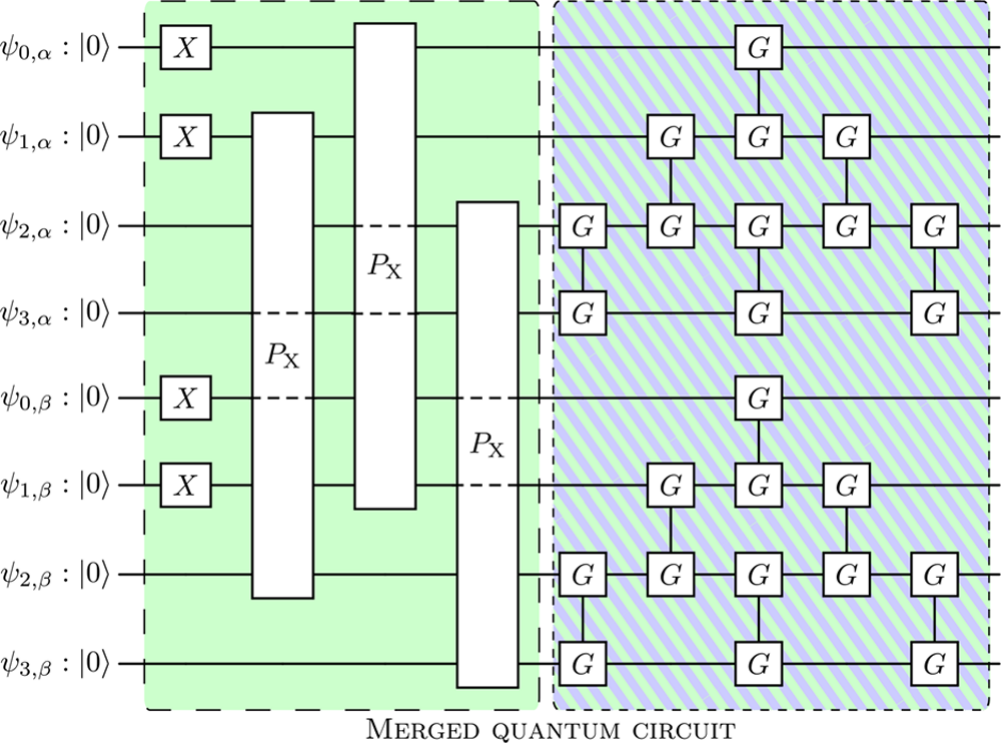
Quantum circuit used in this work. The two-qubit gates labeled with *G* denote Given rotations, which are equivalent to local fermionic basis rotations after Jordan-Wigner mapping. The four-qubit *P*_*X*_ gate represents a PairExchange gate.^27^ We refer to section S4 of the Supporting Information for more details. The Givens rotations of the VQE circuit were merged with the basis rotation circuit (hatched area) to reduce the circuit depth.

To find the ground state energy in the active space, the VQE algorithm^8^ is used. The fermionic Hamiltonian in the (4e, 4o) active space is mapped through the Jordan-Wigner mapping onto an eight-qubit system, in α-then-β ordering, meaning the first (last) four qubits are assigned to the α/spin-up orbitals (β/spin-down orbitals) with a qubit in state |1⟩/|0⟩ representing an occupied/unoccupied orbital. We adapt the VQE ansatz from ref (27) to generate an approximate ground state. This ansatz is composed out of an alternating application of Givens rotations, *G*(θ) (i.e., fermionic basis rotations represented after Jordan-Wigner transformation) and fermionic double excitations, *P*_*X*_(θ), where we restrict our ansatz to a single layer of each, see Figure 3 and section S4 of the Supporting Information. The set of parameters yielding an approximation of the ground state is found by optimizing the expectation value of the second quantized Hamiltonian with the L-BFGS-B algorithm using a classical simulator implemented in Qiskit.^32^ Independent of the number of layers, the last action of the ansatz corresponds to a Givens rotation network. As the electrostatics require only the diagonal part of the 1-PDM γ̃_*vv*_ in the electrostatic potential natural orbital basis, we implement the fermionic basis rotation *U*_*tv*_ that diagonalizes  by another network of Givens rotation gates identical in structure to this last part of the ansatz. We leverage the property that the multiplication of two Givens rotation networks is itself another Givens rotation network; see section S4 of the Supporting Information for details. This allows us to merge the two Givens rotation networks at the end of our circuit and reduce the circuit depth; see Figure 3. We finally measure the qubits in their computational basis to obtain the expectation value of the electrostatics operator in the active space and combine it with the contribution from the core as explained in section S3 of the Supporting Information.

## 4 Hardware Implementation and Results

As quantum hardware, we used the quantum processor *aqt*_*marmot* hosted by Alpine Quantum Technologies GmbH (AQT).^33^ The *aqt*_*marmot* system is based on trapped ^40^Ca^+^ ions and supports a universal set of gates. The native gate set comprises single-qubit gates with arbitrary rotation angles and axis. The entangling operation is a two-qubit Mølmer-Sørensen (MS) gate.^34^ The MS gate allows for entangling operations with arbitrary rotation angles that can be implemented between any qubit pair (see section S4 of the Supporting Information). In this work, we utilize an eight-qubit register featuring all-to-all connectivity. We choose *R*_*X*_(θ) = exp[−*i*θ/2*X̂*_*i*_], *R*_*Z*_(θ) = exp[−*i*θ/2*Ẑ*_*i*_] and *R*_*XX*_(θ) = exp[−*i*θ*X̂*_*i*_*X̂*_*j*_] with *X̂*_*i*_ and *Ẑ*_*i*_ being the Pauli-*X* and Pauli-*Z* matrices acting on qubit *i*, respectively, as the basis gate set since it closely resembles the native gate set of the *aqt*_*marmot* system. The gate errors can be approximated by depolarizing the noise acting on the addressed qubits. The error rates for the single-qubit gates (*R*_*X*_ error) are approximately 3 × 10^–4^ on average, whereas the error rate for the two-qubit gate (*R*_*XX*_ error) is around 1.5 × 10^–2^ on average. The typical gate times are 15 μs for single-qubit gates and 200 μs for two-qubit gates. The *T*_1_ and *T*_2_ times are 1.14 ± 0.06 s and 0.452 ± 0.068 s, respectively, resulting in a coherence/gate time ratio of about 10^3^.^35^

We used the quantum circuit described in section 3 to prepare an approximate ground state on the quantum computer. We compile the quantum circuits’ gates into the hardware’s native gate set to implement the circuit on the trapped-ion quantum computer. Subsequently, we use Qiskit’s transpiler^32^ to further optimize the circuit and minimize the total number of gates. After optimization, our circuit consists of 279 single-qubit gates and 63 two-qubit gates. The transpiled circuit for intermediate A is displayed in section S4 of the Supporting Information.

**Figure 4:**
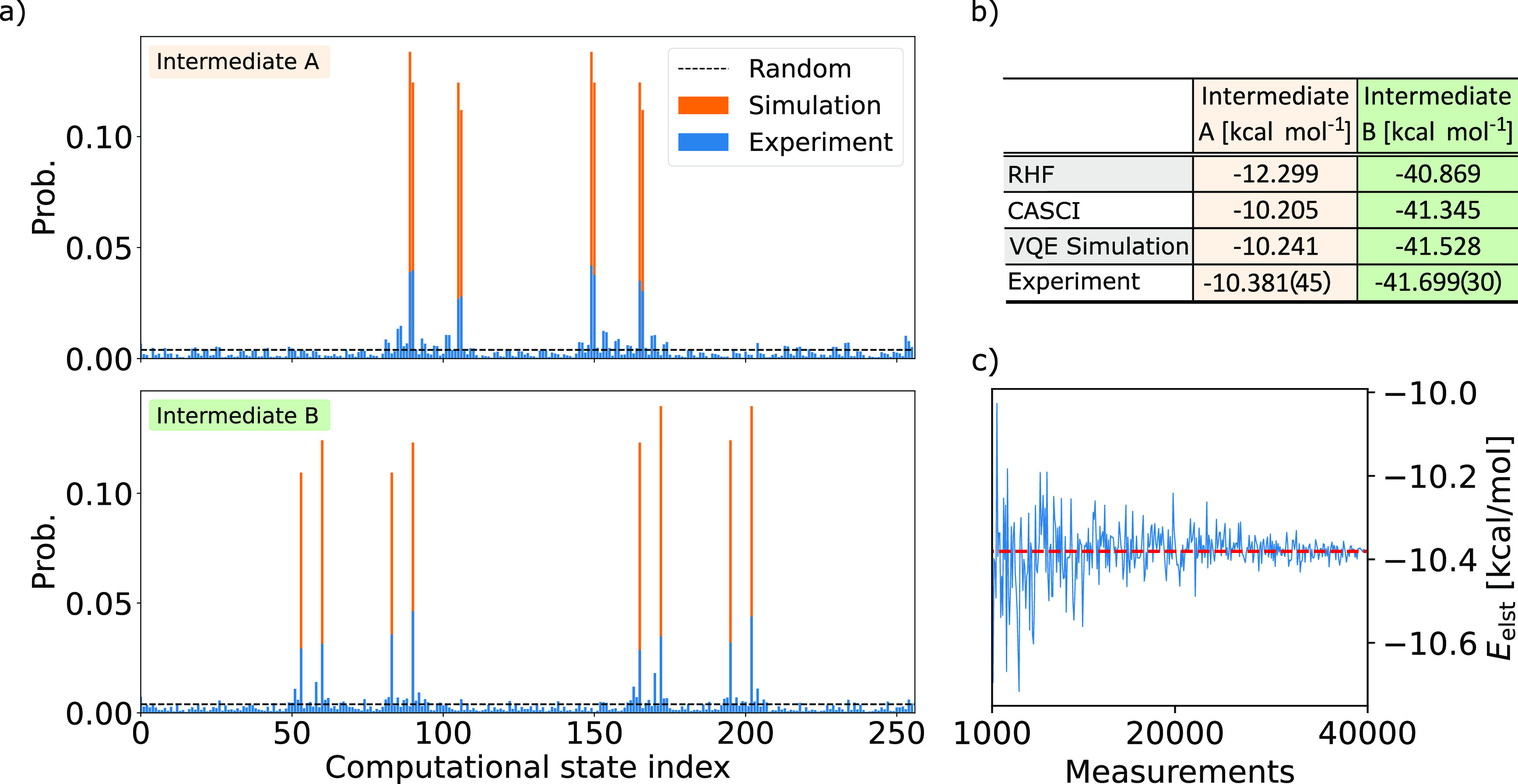
(a) The output statistics of the VQE circuit, including the final basis rotation for intermediate A (top) and intermediate B (bottom). The experimental raw outcome (blue) is compared to exact classical simulation results (orange) and random sampling (black line). For each experiment, *N*_meas_ = 4 × 10^4^ measurements were used. (b) A comparison between the electrostatic energies obtained from various classical and quantum methods. For the experimental results, the raw data were postselected on the correct particle number before calculating the electrostatics energies. (c) The convergence of the electrostatics of intermediate A as a function of the number of measurements used to construct the 1-PDM in eq 12.

In Figure 4a, we present the output statistics obtained from *N*_meas._ = 4 × 10^4^ measurements for intermediates A and B. We compare the experimental results to results from an error-free VQE simulation on a classical computer. We also put these distributions in contrast with the uniform distribution (random sampling). Despite a non-negligible level of noise, we observe strong agreement in the dominant computational basis states. To obtain the electrostatic interaction energies for both intermediates, we first exclude all computational states with incorrect α and β particle numbers from the measured data, a technique commonly referred to as *postselection*. In both experiments, we discard roughly 50% of the samples. In section S5 of the Supporting Information, we plot the output statistics after the postselection. We then construct the diagonal parts of the rotated 1-PDM using eq 12 and compute the electrostatics according to eq 11. We summarize our findings in Figure 4b. We find that the electrostatic energies of both systems are within chemical accuracy (1 kcal mol^–1^) to the error-free VQE simulations, with deviations of Δ*E* = −0.204(45) kcal mol^–1^ and Δ*E* = −0.354(30) kcal mol^–1^ for intermediate A and intermediate B, respectively. To estimate the statistical errors, we calculate the electrostatic energy for each measurement with the correct particle number separately. From the calculated *N*_cor_ different values of the electrostatics, we determine the standard error of the mean, given by , where σ is the standard deviation of the list and *N*_cor_ is the number of measurements with correct particle number. In Figure 4c, we also report the convergence of the electrostatics when the number of measurements is increased to construct the required 1-PDM. Here, for clarity, we report estimates obtained from using more than 1000 measurements.

In section S5 of the Supporting Information, we show the same convergence results using 2 to 1000 measurements. In this case, the calculated electrostatic energies lie in a much broader range (from −26.98 kcal mol^–1^ to 6.80 kcal mol^–1^). This emphasizes that the spectrum of our estimate is nontrivial but that its expectation value converges within thousands of measurements, a number feasible on the quantum hardware used. This also indicates that the remaining Δ*E* is a consequence of device noise biasing our estimate. In section S5 of the Supporting Information, we also explore results from random sampling, which yields fairly accurate electrostatic energies. By comparing the output statistics between experimental results, noise-free simulations, and random sampling, we argue that the observed phenomenon is an attribute of the system under investigation and does not undermine the experimental outcome.

From the experimental results, we calculate a difference in electrostatic energies of Δ*E*_elst_^exp^ = −31.32(5) kcal mol^–1^ between intermediate A and B, allowing us to answer the question raised in section 3 and conclude that the electrostatics favors the stabilization of NADH in the heme pocket. Although, for this model system, this could have been qualitatively described using Hartree–Fock theory (Δ*E*_elst_^RHF^ = −28.57 kcal mol^–1^), our results quantitatively align with CASCI calculations (Δ*E*_elst_^CASCI^ = −31.14 kcal mol^–1^).

Multiple efforts to reduce the noise bias Δ*E* were explored: To investigate and mitigate potential biases stemming from keeping the ion-qubit assignment fixed during quantum computation, we performed additional experiments, where we introduced a random alteration of the qubit-ion assignment every 100 experimental runs. Additionally, to mitigate the impact of qubit decay from |1⟩ → |0⟩, we treated the qubits’ zero state |0⟩ to represent an occupied orbital, while |1⟩ represents an unoccupied orbital. This can be easily implemented by adding a full layer of Pauli-*X* gates at the beginning of the quantum circuit while concurrently substituting all circuit parameters with their negated values. As demonstrated in section S5 of the Supporting Information, incorporating these error mitigation techniques does not significantly improve the accuracy of the electrostatic energy. We also classically simulated the effect of Zero-Noise-Extrapolation (ZNE), an error mitigation method well-suited for depolarizing errors as they predominate in the used quantum processor (see section S5 of the Supporting Information). While we found an improvement of the electrostatic energy, the error of the estimate increased significantly. To reduce the error, a substantial increase in the number of experimental measurements would have been necessary.

In order to draw a comparison between our approach and the supermolecular approach given in eq 1, we would require estimation of the ground state energies of both monomers and the larger dimer system. However, since we only measure the diagonal elements of the rotated 1-PDM of monomer A, as per eq 12, we cannot use these measurements to estimate the ground state energy of monomer A, as this would necessitate the full 1-PDM and the 2-PDM. Instead, we use these measurements to compute the expectation value of the diagonal elements of the one-body part of the Hamiltonian, with further details provided in section S6 in the SI. A deviation of 1.16 kcal mol^–1^ was observed when comparing the experimental results with the noise-less simulation. Considering the error in the diagonal contribution to the one body energy, it is unlikely that the accuracy of the ground state energies of the monomer systems would be sufficient to estimate interaction energies within chemical accuracy, let alone the ground state energy of the larger dimer system.

## 5 Conclusion

In our study, we performed the first direct quantum computations of interaction energies; specifically, we computed the electrostatic energies of two important intermediates in the catalytic conversion of NO to N_2_O by P450nor. Using a VQE ansatz, we generated an approximate ground state for one of both monomers on a trapped-ion quantum computer, while the other was treated with classical HF theory. A fermionic basis rotation at the end of the quantum computation allowed the efficient measurement of the electrostatics energy with a single quantum circuit and without extending the circuit length. We found that the quantum-computed electrostatics strongly agree with our simulation results, within a chemical accuracy of 1 kcal mol^–1^. As measuring under these fermionic basis rotations is also used when, e.g., measuring ground state energies efficiently with double factorization,^9,21,22^ this experiment implies that these techniques are starting to be in reach of current hardware.

In order to execute the demonstrated quantum experiment, we had to considerably simplify the computational problem to conform to a limited number of qubits, coherence times, and gate fidelities available on the quantum computer. Furthermore, we employed classical simulations of the quantum circuit to optimize the variational parameters. This allowed us to leave aside the task of finding the ground state in a noisy environment and focus our study solely on the computation of the electrostatic energy. While this work does not offer new insights into the systems’ chemistry, it does provide the first experimental demonstration of the potential usefulness of quantum algorithms that are tailored to the computation of quantities beyond ground state energies. In particular, we showed how the measurement count can be reduced in practice, resulting, for the small models considered, in a shot noise resilient estimate.

Scaling our approach to industry-relevant problems requires further research. First, the electrostatic energy is only a single term of SAPT, which by itself does not require symmetry adaption. Obtaining other terms such as the exchange, induction, and dispersion is vital to accurately describe the interaction between two molecules. To this end, efficient measurement strategies similar to those in this work must also be developed. For example, when computing the exchange energy, one could represent the required quantities as sum of diagonal operators supplemented by basis rotations,^19,36^ allowing again for postselection of the measurement data on the particle number. Another crucial aspect for future research resides in finding efficient measurement schemes when both monomers exhibit strong correlations and require accurate descriptions of quantum computers.

While tailored quantum computations, such as the one presented, allow pushing the boundaries of existing quantum hardware, it is unlikely that a quantum advantage will become possible without exploiting any form of error mitigation or limited error correction. Fault-tolerant quantum computations of SAPT energies^37^ could potentially offer another approach to the quantum computation of interaction energies.

## Data Availability

The presented measurements were performed on the full-stack quantum computer *aqt_marmot*. We would like to point out that this particular device is no longer online and available, but it was replaced with a new system hosted by AQT. The measured data are available from the corresponding author upon reasonable request.

## Supporting Information Available

Supporting text, Figures S1–S10, Tables S1 and S2, and references (PDF)
