# Supplementary material for: Estimation of Electrostatic Interaction Energies on a Trapped-Ion Quantum Computer
Source: ACS Cent Sci. 2024 Mar 26;10(4):882–9. doi: 10.1021/acscentsci.4c00058 (PMC11046474; doi:10.1021/acscentsci.4c00058)
Supplement: Supplementary file 1 — oc4c00058_si_001.pdf [file oc4c00058_si_001.pdf]

Supporting information for
Estimation of electrostatic interaction energies on a trapped-ion quantum computer

Pauline J. Ollitrault ,* Matthias Loipersberger , and Robert M. Parrish
QC Ware Corp, Palo Alto, USA and Paris, France

Alexander Erhard , Christine Maier , Christian Sommer , Juris Ulmanis , and Thomas Monz †
Alpine Quantum Technologies GmbH, 6020 Innsbruck, Austria

Christian Gogolin
Covestro Deutschland AG, 51373 Leverkusen, Germany

Christofer S. Tautermann ‡
Medicinal Chemistry, Boehringer Ingelheim Pharma GmbH & Co. KG, 88397 Biberach, Germany

Gian-Luca R. Anselmetti, Matthias Degroote , Nikolaj Moll , Raffaele Santagati , and Michael Streif §
Quantum Lab, Boehringer Ingelheim, 55218 Ingelheim am Rhein, Germany

(Dated: 2023)

CONTENTS

S1. Expression of the electrostatics in terms of the 1-PDM S2

S2. Preparation of intermediates A and B S2

S3. Active space description of electrostatics S5

S4. Quantum circuits S5

S5. Additional experimental results S6

S6. Calculation of the diagonal one-body Hamiltonian energy contribution S8

S7. Error mitigation using Zero-Noise-Extrapolation S10

References S11

This file includes:
Supporting text
Figures S1-S10
Tables S1-S2
SI References

* Email: pauline.ollitrault@qcware.com
† Also at Universität Innsbruck, Institut für Experimentalphysik, Innsbruck, Austria
‡ Also at University of Innsbruck, Department of General, Inorganic and Theoretical Chemistry, Innsbruck, Austria
§ Email: michael.streif@boehringer-ingelheim.com

S1. EXPRESSION OF THE ELECTROSTATICS IN TERMS OF THE 1-PDM

In this section we formally define the electrostatic energy in terms of the spin-summed one-particle density matrix (1-PDM) which, in spatial coordinates is,

$$\gamma(\vec{r}_1, \vec{r}_{1'}) \equiv \int \mathrm{d}\sigma_1 \int \mathrm{d}\sigma_{1'} \int \mathrm{d}\vec{x}_2 \ldots \int \mathrm{d}\vec{x}_N \; \Psi(\vec{x}_1, ..., \vec{x}_N)\Psi(\vec{x}_1, ..., \vec{x}_N) \equiv \sum_{pp'} \gamma_{pp'}\phi_p(\vec{r}_1)\phi_{p'}(\vec{r}_{1'}) \tag{S1}$$

where $\gamma_{pp'}$ is the spin-summed 1-PDM in a (non-orthogonal) spin-restricted spatial orbital basis $\{\phi_p(\vec{r})\}$.

In this notation, the electrostatic energy can be written as

$$E_{\mathrm{elst}} = \sum_{pp'}\sum_{qq'} \gamma^A_{pp'}(pp'|qq')\gamma^B_{qq'} + \sum_{pp'} \gamma^A_{pp'} V^B_{pp'} + \sum_{qq'} V^A_{qq'}\gamma^B_{qq'} + V_{AB}. \tag{S2}$$

Here, $\gamma^A_{pp'}$ is the 1-PDM of monomer A. The two electron integrals are defined as

$$(pp'|qq') = \int_{\mathbb{R}^6} \mathrm{d}^3\vec{r}_1 \mathrm{d}^3\vec{r}_2 \; \phi_p(\vec{r}_1)\phi_{p'}(\vec{r}_1)\frac{1}{r_{12}}\phi_q(\vec{r}_2)\phi_{q'}(\vec{r}_2). \tag{S3}$$

The nuclear potential, $V^A_{pp'}$ of monomer A is

$$V^A_{pp'} = -\sum_{\mathcal{A}}^{M_A} \int_{\mathbb{R}^3} \mathrm{d}\vec{r}_1 \; \phi_p(\vec{r}_1)\phi_{p'}(\vec{r}_1)\frac{Z_{\mathcal{A}}}{r_{1\mathcal{A}}} \tag{S4}$$

where M_A is the number of atoms in monomer A and $Z_{\mathcal{A}}$ is the nuclear charge of atom $\mathcal{A}$. Finally, the inter-monomer nuclear repulsion is given by

$$V_{AB} = \sum_{\mathcal{A}}^{M_A}\sum_{\mathcal{B}}^{M_B} \frac{Z_{\mathcal{A}}Z_{\mathcal{B}}}{r_{\mathcal{AB}}}. \tag{S5}$$

Eq. (S2) and Eq. (3), in the main text, are easily related by noting that the number of electrons in monomer A is

$$N_A = \sum_{pp'} S_{pp'}\gamma^A_{pp'} \tag{S6}$$

where $S_{pp'}$ is the overlap matrix,

$$S_{pp'} \equiv \int_{\mathbb{R}^3} \mathrm{d}^3\vec{r}_1 \; \phi_p(\vec{r}_1)\phi_{p'}(\vec{r}_1). \tag{S7}$$

S2. PREPARATION OF INTERMEDIATES A AND B

We extract models for intermediates A and B from highly resolved crystal structures taken from the protein data bank (PDB). The PDB entries are 1CL6 [1] and 1XQD [2] for intermediate A and B, respectively. Both structures are first subjected to the Protein Structure Preparation Procedure as implemented in MOE [3], which adjusts hydrogens, partial charges, non-modeled side chains, and hybridizations. Our focus is on the active site. Therefore we prepare model systems that contain most of the important structural features around the heme binding site. The heme iron is coordinated to Cys352 from below, and we truncate this ligand to a methylthiolate ion to resemble the electronic effects of cysteine on the iron. All side chains are removed from the heme itself, as they do not interfere with the binding site. On the NO-binding site, we keep the three amino acids Ala239, Thr243, and Ser286 as these residues are strongly involved in a hydrogen bond network including waters and NADH (if present). The three amino acids are kept in their respective orientations from the PDB files, the amide bonds along the protein backbone are cut, and the rest of the protein is removed. To maintain the correct chemical environment of the three amino acids, the termini are capped to resemble amide bonds (acetylation of the N-terminus and amidation of the C-terminus), thereby ensuring the correct amide bond orientation according to the X-ray structure. In the model of intermediate A, the NO ligand bound to the heme and additional three water molecules (WAT548, WAT576, WAT794 in 1CL6, as displayed in Figure S2) are included as they form a strong hydrogen bond network within the three amino acids and the NO

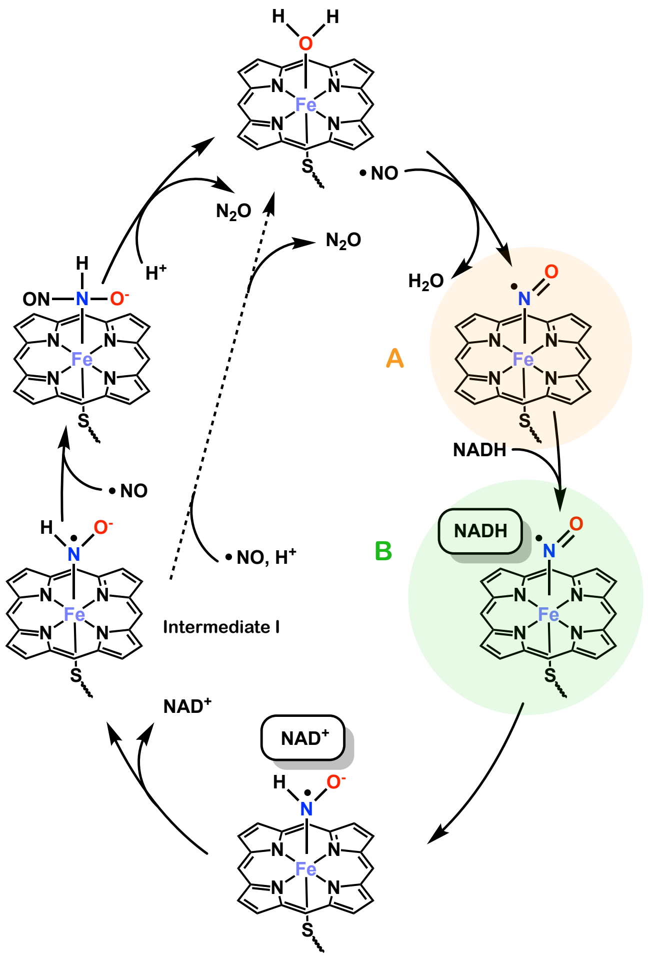

FIG. S1. Reaction scheme of the reduction of NO to N_2O catalysed by P450nor. In our study we focus on NO-bound intermediates A and B and the question whether the electrostatic interaction favour the stabilisation of the NADH molecule in the heme pocket.

ligand. In the model of intermediate B, additional steps are required to model the active site. A water (WAT565) from the X-ray structure, hydrogen-bonded to Thr243 is included. The carboxylic acid group is modified to a carboxamide group (as in NADH) so that the carbonyl group still forms a hydrogen bond to the backbone NH from Ala239. The pyridine ring is reduced by adding a hydrogen atom (to have the reduced form: NADH), and the ligand is capped after the first ribose-carbon. The rest of the NADH is assumed not to contribute to the hydride-transfer reaction. The NO-ligand is manually put to the heme to finalize the model system with all relevant and correct species. Then, a force field optimization of the hydrogen placements in MOE (AMBER10:EHT) is done, followed by a tethered minimization (tether on all heavy atoms). An overlay of the two resulting model structures is shown in Fig. S2.

Finally, both structures are optimized with density functional theory (DFT). The position of the heme is held fixed and the position of the C_α atoms of the amino acids is also constrained as a surrogate for the protein backbone environment that keeps amino acid side chains and co-factors in place. We use the GPU accelerated DFT implementation of Promethium [4] on a single A100 GPU, allowing us to perform the full geometry optimization at B3LYP-D3/def2-SV(P) level of theory in 3h58 for intermediate A (1042 basis functions) and 6h36 for intermediate B (1130 basis functions). The resulting geometries are shown in Fig. S3.

Because we are limited quantum resource available on current hardware, we devise an active space of four molecular orbitals (MOs). The size of this active space is solely chosen to fit the hardware constraints and does not reflect the important chemistry in our systems. Therefore, we choose to employ an automated active space selection approach rather than picking the orbitals fully based on their symmetries. To do so, we first run a semistochastic heat bath configuration interaction (SHCI) (as implemented in Dice [5, 6]) for the two systems in the singlet state with $\epsilon = 0.01$, the def2-SVP basis set, in a 20 electrons in 20 orbitals (taken around the HOMO-LUMO gap). This starting active

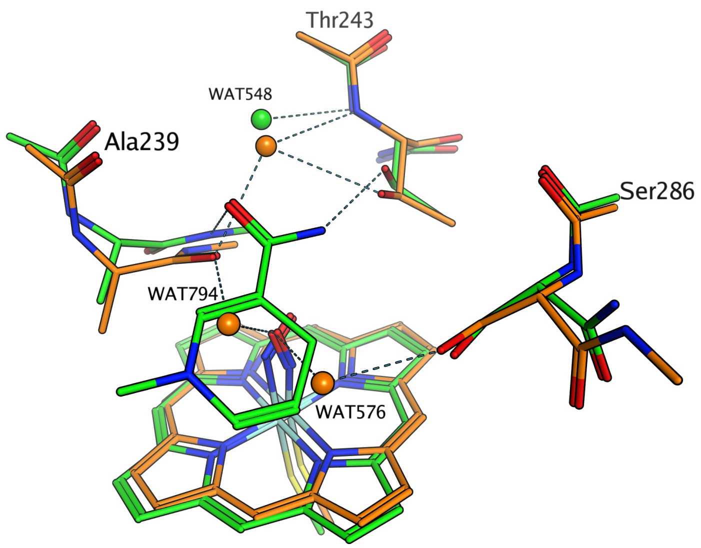

FIG. S2. Overlay of the model complexes before DFT optimization. Intermediate A and B are shown in orange and green, respectively. Dashed lines denote hydrogen bonds and the water labels correspond to pdb entry 1CL6.

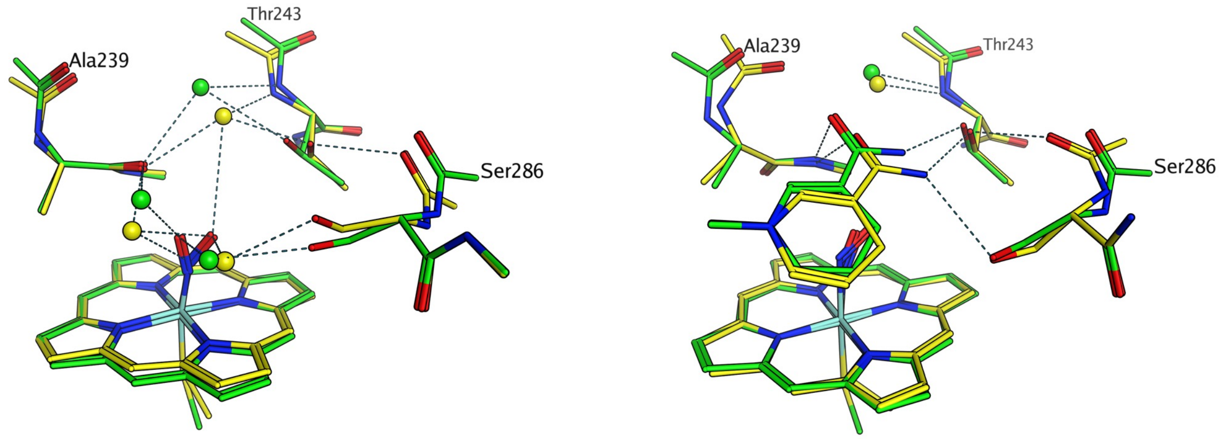

(a) Intermediate A

(b) Intermediate B

FIG. S3. Overlay of the structures of both intermediates before (green) and after (yellow) geometry optimization via DFT. Dashed lines denote hydrogen bonds.

space is sizeable enough to include the iron 3d, iron 4s, iron-nitrogen anti-bonding and axial ligand (anti-)bonding orbitals [7]. From the resulting 1-PDM, we obtain the natural orbitals and order them according to their occupation numbers. We show the four orbitals around the gap in Fig. S4(left) along with their occupation numbers. These orbitals are a mixture of the iron 3d, the iron-nitrosil (anti)bonding π, the sulfur p as well as heme π orbitals which are all important to capture the essential mixing. Hence, as a final step we run a complete active space self-consistent field (CASSCF) calculation with PySCF [8] using the SHCI natural orbital as a starting guess and a 4 electrons in 4 orbitals active space (4e, 4o). The resulting orbitals define our final (4e, 4o) active space. They are displayed in Fig. S4(right). In both intermediates, two orbitals show strong deviation from integer values indicating the multi-reference character of the systems and exhibit the expected metal ligand backbonding of Fe and NO and porphyrin π and π^* orbitals. Note that the inclusion of the first and last orbitals in the active space (the one with occupation numbers close to 2 and 0, respectively) lowers the total energy by 18 mHa for intermediate A and 10 mHa for intermediate B, justifying their importance in the ground state.

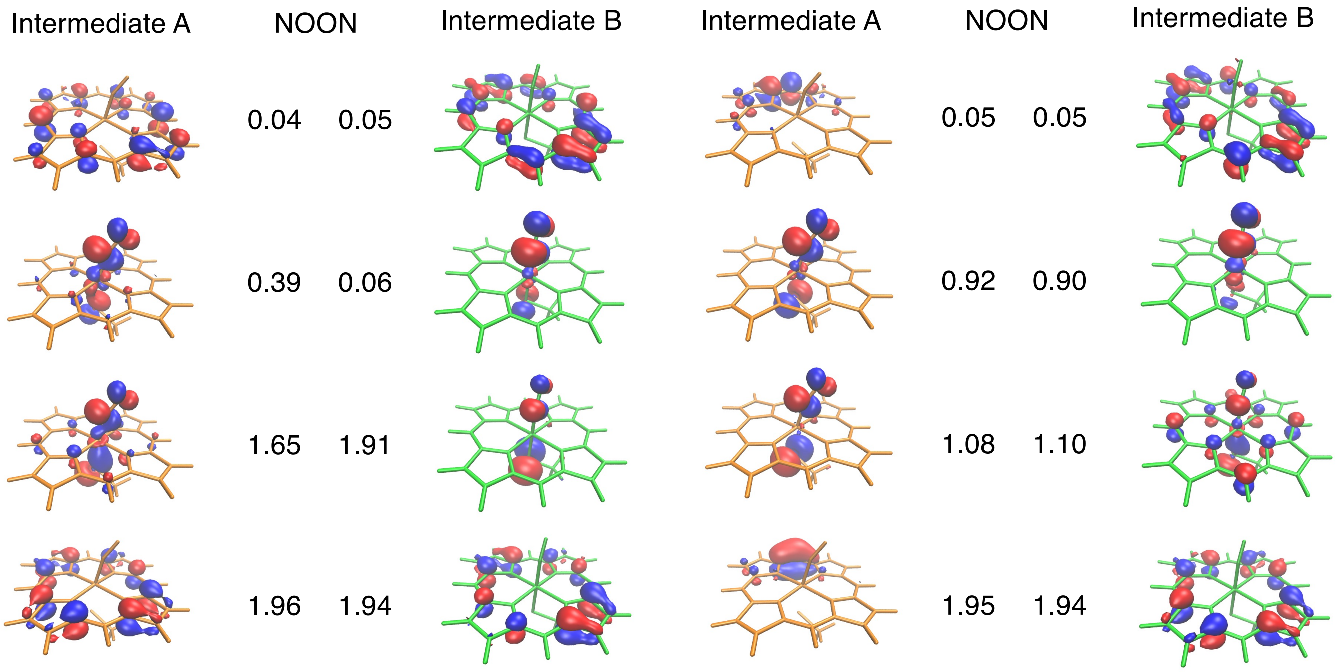

FIG. S4. (left) The four SHCI natural orbitals around the gap and their occupation numbers, (right) The four CASSCF molecular orbitals around the gap and their occupation numbers

S3. ACTIVE SPACE DESCRIPTION OF ELECTROSTATICS

In the following, we describe how to recover the electrostatic energy in the case where the quantum computer is used to generate an approximate ground state in an active space.

Since the 1-PDM is block diagonal, we can rewrite Eq. (8) from the main text as

$$E_{\text{elst}} = \sum_{t,t'=0}^{N_{\text{core}}} \mathcal{J}^B_{tt'}\gamma^A_{tt'} + \sum_{t,t'=N_{\text{core}}}^{N_{\text{act}}} \mathcal{J}^B_{tt'}\gamma^A_{tt'} \tag{S8}$$

where in the first (second) term the indices run over the MOs in the core (active) space. In the core space, by definition $\gamma^A_{tt'}$ is diagonal (with 2s on its diagonal). Hence, we only rotate the orbital basis in the active space and, as per Eq. (11), we find

$$E_{\text{elst}} = 2\sum_{t=0}^{N_{\text{core}}} \mathcal{J}^B_{tt} + \sum_{v=N_{\text{core}}}^{N_{\text{act}}} \bar{w}_v \bar{\gamma}^A_{vv}. \tag{S9}$$

S4. QUANTUM CIRCUITS

The VQE circuit was designed on the model of the quantum number preserving ansatz of Ref. [9]. This ansatz is composed out of an alternating application of Givens rotations, $G(\theta)$ (i.e. fermionic basis rotations represented after Jordan-Wigner transformation) and fermionic double excitations, $P_X(\theta)$. To maximally reduce the circuit depth while keeping a general architecture, ignoring a priori knowledge of the targeted ground state, we follow three steps. First, we keep only one layer of each kind ($G(\theta)$ and $P_X(\theta)$). Second, in both layer kinds, we start by entangling the last occupied and first unoccupied qubits (as defined in the initial Hartree-Fock (HF) state). We then progressively entangle the next two qubits up and down, in a ladder way, until we reach to first and last qubit, respectively. In our small 4+4 qubit system this only translates to 3 $P_X(\theta)$ and 3+3 $G(\theta)$ gates. Third, we first add the $P_X(\theta)$ layer and then the $G(\theta)$ layer. This setup allows us to merge the $G(\theta)$ layer with the subsequent Givens rotation network, representing the fermionic basis rotation into the electrostatic potential natural orbital basis, shown in Sec. II in

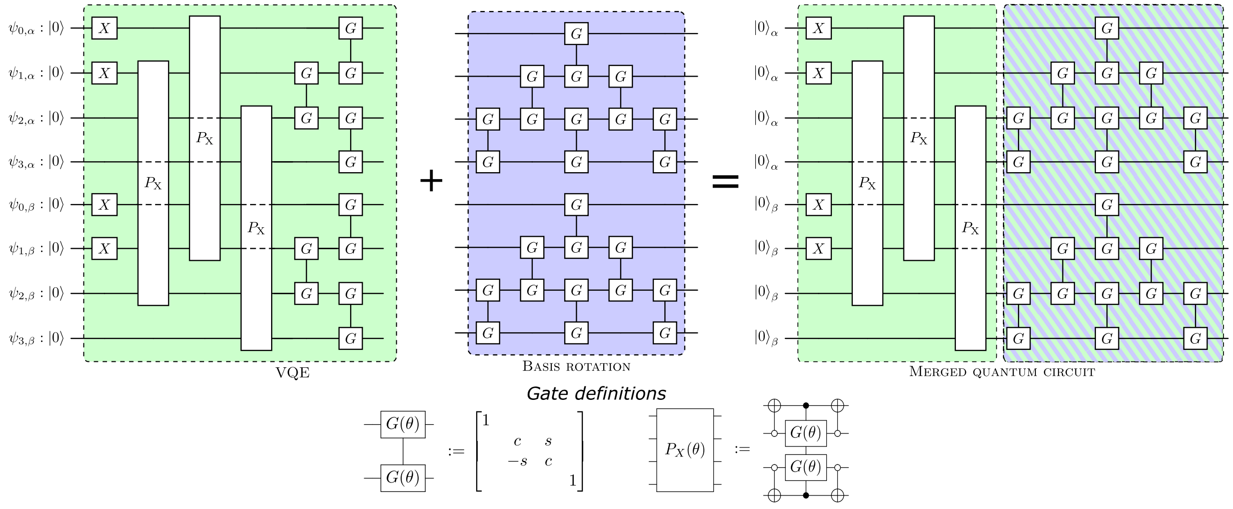

FIG. S5. (top) The quantum circuit used in this work. The two-qubit gates labeled with G denote Given rotations, which are equivalent to local fermionic basis rotations after a Jordan-Wigner mapping. The four-qubit P_{X} gate represents a PairExchange gate, see below. The green circuits represent the VQE ansatz, while the blue circuit represents the basis rotation to efficiently measure the electrostatic interaction. On the right hand side, the Givens rotations network of the VQE circuit was merged with the basis rotation circuit (hatched area). (bottom) Gate definitions of the Givens rotation gate $G(\theta)$ and the PairExchange Gate $P_X(\theta)$ as introduced in [9]. Matrix entries are $c := \cos(\theta/2), s := \sin(\theta/2)$.

the main text. We use the fact that two Givens rotation network can be easily merged. As every Givens rotation network represents a fermionic basis rotation U_{tv}, two Givens rotation networks can be easily merged by multiplying the underlying fermionic basis rotations. From the found new basis rotation, we generate a new Givens rotation network. The circuit architecture together with the merging of the Givens rotation networks is more clearly depicted on Fig. S5.

As explained in the main text, we choose the gates $R_{\mathrm{X}}(\theta), R_{\mathrm{Z}}(\theta), R_{\mathrm{XX}}(\theta)$ as target gate set for the Qiskit transpiler, where the $R_{\mathrm{XX}}(\theta)$ entangling operation is a two-qubit Mølmer-Sørensen (MS) gate that can also be described as,

$$U_{\mathrm{MS}}(\theta) = \begin{pmatrix} c & 0 & 0 & -\mathrm{i}s \\ 0 & c & -\mathrm{i}s & 0 \\ 0 & -\mathrm{i}s & c & 0 \\ -\mathrm{i}s & 0 & 0 & c \end{pmatrix}, \tag{S10}$$

with $c = \cos(\theta/2)$ and s=sin $(\theta/2)$.

In Figure S6 and Figure S7 we show the full transpiled quantum circuit used to compute the electrostatic energy in intermediate A. The circuit comprises a total number of gates of $(R_{\mathrm{Z}}, 168), (R_{\mathrm{X}}, 111), (R_{\mathrm{XX}}, 63)$. The circuit for intermediate B is similar to the circuit for intermediate A with a total number of gates of $(R_{\mathrm{Z}}, 168), (R_{\mathrm{X}}, 113), (R_{\mathrm{XX}}, 63)$.

Note that for both systems, the optimized angles of the last two $P_X(\theta)$ gates are small ($\approx 10^{-3}$). Their contribution to the energy is therefore negligible here. However, the standard Qiskit transpiler does not do circuit approximation, which is why we implement every circuit unitary as specified. In principle, the circuit size could be further minimized using approximation methods, where the level of approximation would depend on the noise of the specific hardware. While it seems trivial for a 8 qubit circuit with few gates, this emphasizes the need for robust transpilation codes or for guided circuit construction techniques [10] when scaling up to relevant size systems. Here, in the pursuit of reporting the performance of state of the art quantum hardware and software kit, we keep the resulting transpiled circuit while bearing in mind that the noise introduced by applying the previously mentioned $P_X(\theta)$ gates will probably be greater than their contribution to the energy.

S5. ADDITIONAL EXPERIMENTAL RESULTS

The electrostatic energy of our model system, constrained to a small active space to align with experimental constraints, exhibits minimal variation in response to state errors. To validate the reliability of our results as a consequence of successful quantum computation rather than mere chance, we present additional experimental data below. In Table S1, we first compare the electrostatic energies obtained in classical simulation of an exact superposition

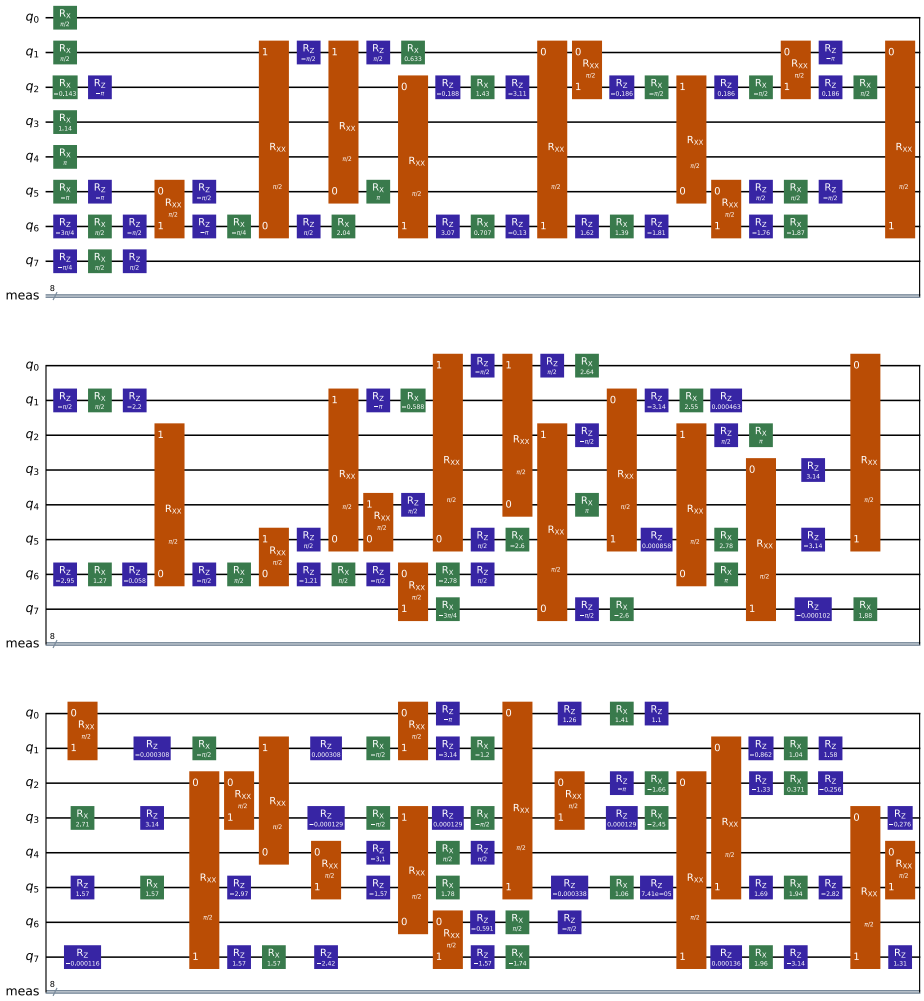

FIG. S6. Part 1 of transpiled quantum circuit for intermediate A. Rx, Rz and Rxx gates are shown in green, blue and orange, respectively.

state (random sampling) and of the VQE state. The difference between the two intermediate, $\Delta E_{\mathrm{elst}} = E_{\mathrm{elst}}(\mathrm{B}) - E_{\mathrm{elst}}(\mathrm{A})$, is -31.751 kcal mol^{-1} for random sampling, -31.314 kcal mol^{-1} for the exact VQE. These values are to be compared to the -31.140 kcal mol^{-1} predicted by CASCI. While the VQE results bring us closer to CASCI, the random sampling outcomes also fall within chemical accuracy, making it challenging to definitively conclude the true success of the experiment from looking exclusively at the electrostatic energy. For this reason, in Table S2, we illustrate the correspondence between the statistics derived from the noise-less simulation of the quantum circuit and both the experimental results and the exact superposition distribution associated with random sampling. Here, the consistency of the results becomes more evident, with the overlaps of the experimental distributions significantly surpassing those obtained from random sampling. Moreover, in order to determine whether the results using random sampling are systematic or merely coincidental to the systems under study, we compute the electrostatics energy using CASSCF and random sampling on a water dimer system in a (4,4) active space using a 6-31g basis and the geometry from [11]. We find a discrepancy of approximately 4 kcal mol^{-1} between the two methods. Interestingly, this gap widened to nearly 7 kcal mol^{-1} when we expanded the active space to (8,8). We therefore conclude that this occurrence is likely a system-specific coincidence, potentially also influenced by the constraints of the limited active space used.

For each intermediate, we also present 4 different experiments: two solely derived from the VQE quantum circuit (plain), and the remaining two incorporating error mitigation techniques (mitigated). In particular, we aim to mitigate potential biases coming from the difference in qubit performance and from qubit relaxation ($|1\rangle \rightarrow |0\rangle$). Therefore, in these additional experiments, we introduced a random alteration of the qubit-ion assignment every 100 experimental runs along with an inversion of the qubits' $|0\rangle$ and $|1\rangle$ states to represent an occupied orbital and an unoccupied orbital respectively. This last bit can be easily implemented by adding a full layer of Pauli-X gates in the beginning

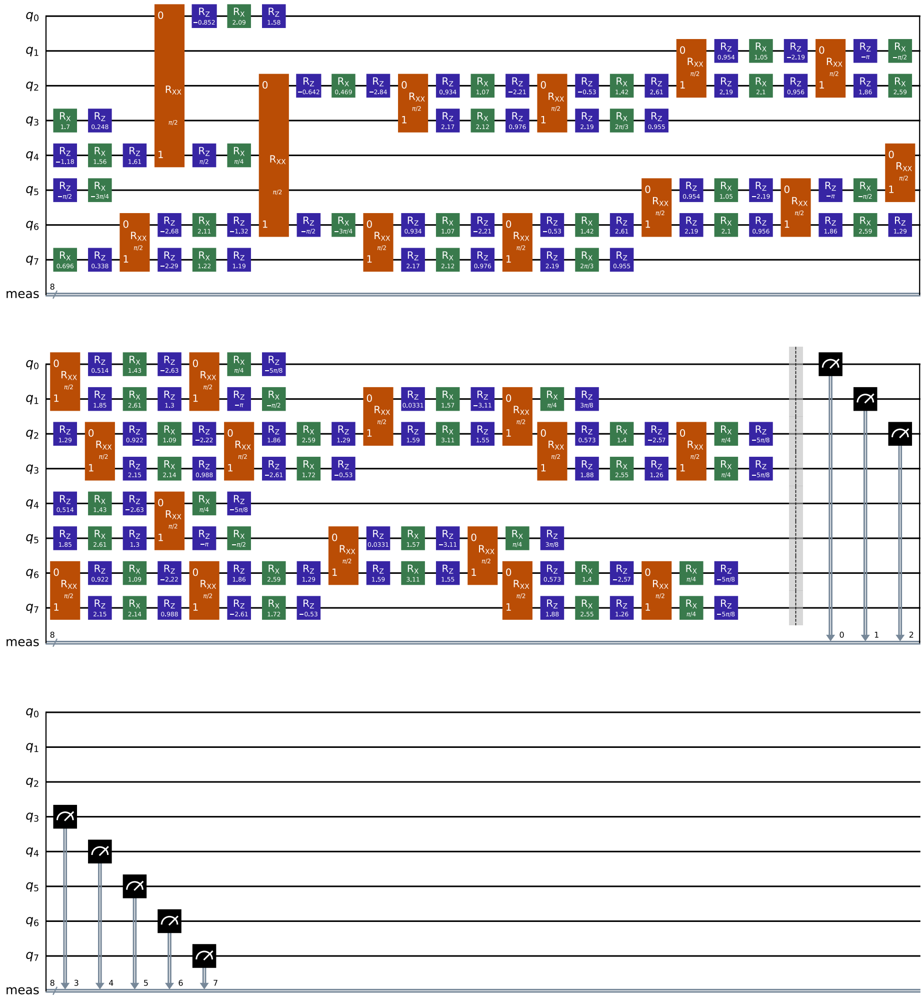

FIG. S7. Part 2 of transpiled quantum circuit for intermediate A. Rx, Rz and Rxx gates are shown in green, blue and orange, respectively.

of the quantum circuit while concurrently substituting all circuit parameters with their negated values.
The maximum absolute deviation in the resulting four electrostatic energies from the expected exact VQE results is very low, 0.293 kcal mol^{-1} for intermediate A and 0.341 kcal mol^{-1} for intermediate B. This demonstrates the concurrence of our results. Nevertheless, it is noteworthy that no discernible improvement is observed with the implementation of error mitigation protocols.

For completeness, in Fig S9, we report the experimental data after post-selecting on the correct particle numbers. Finally, in Fig. S8, we report the convergence of the electrostatics when increasing the number of measurements to construct the required 1-PDM using from 2 to 1000 measurements. This is similar to Fig. 4(c) of the main text but with a focus on the first thousand measurements. As clearly visible, at low number of measurements, the spread of values is much higher. We note that the lowest/highest possible value of electrostatics energy within the space of computational states with correct particle sector for intermediate A is -26.98 kcal mol^{-1} and 6.80 kcal mol^{-1} respectively. This indicates that the spectrum of E_{elst} in the computational basis states is wide and that, although the average of all of them (cf. Random) takes us close to the right value, in general, a state with the wrong structure would lead to an inaccurate E_{elst}.

S6. CALCULATION OF THE DIAGONAL ONE-BODY HAMILTONIAN ENERGY CONTRIBUTION

To gauge the quality of our results in comparison to the supermolecular approach, we would require ground state energy calculations on the monomer and dimer systems. To estimate the energies, we would require access to the full

	Intermediate A		Intermediate B	
Random	-10.096		-41.847	
VQE sim.	-10.214		-41.528	
Exp. run	Plain	Mitigated	Plain	Mitigated
1	-10.381(45)	-10.409(42)	-41.699(30)	-41.392(29)
2	-10.507(42)	-10.437(43)	-41.733(30)	-41.466(30)

TABLE S1. This table provides an overview over the experiments ran for this study. The labeling plain corresponds to the basic setup described in the main text of this work. The experiments with the label Relabeling exploited a change of the qubit-ion assignement after each 100 shots. Moreover in these experiments, the labeling of 1 and 0 was exchanged. All energies are given in kcal mol^{-1}.

	Intermediate A		Intermediate B	
Random	0.191		0.198	
Exp. Run	Plain	Mitigated	Plain	Mitigated
1	0.544	0.561	0.554	0.547
2	0.573	0.546	0.547	0.572

TABLE S2. Overlap between the experimental runs and the noise-less simulation of the quantum circuit. The overlap was calculated via the Bhattacharyya distance, $BC(\mathbf{p},\mathbf{q}) = \sum_i \sqrt{p_i q_i}$, where p_i and p_i represent the frequencies of the bitstring i from the experimental results and the simulation respectively.

1- and 2-PDMs. However, we only have measured the diagonal parts of the rotated 1-PDM, see Eq. (12) in the main text. As a proxy, we compute the expectation value of the diagonal parts of the one-body Hamiltonian when rotated into the electrostatic potential natural orbital basis. Applying the rotations U_{tv} yields

$$\bar{h}^A_{vv'} = \sum_{tt'} U_{vt} h^A_{tt'} U_{t'v'} \tag{S11}$$

We then use the the measured 1-PDM from Eq. (12) to calculate the expectation value of the diagonal terms

$$E_{\mathrm{diag}(\bar{h})} = \sum_{v} \bar{h}^A_i \bar{\gamma}^A_{vv} \,. \tag{S12}$$

As reported in the main text, we find an error of 1.16 kcal mol^{-1}, which is significantly larger than the error found for the electrostatics.

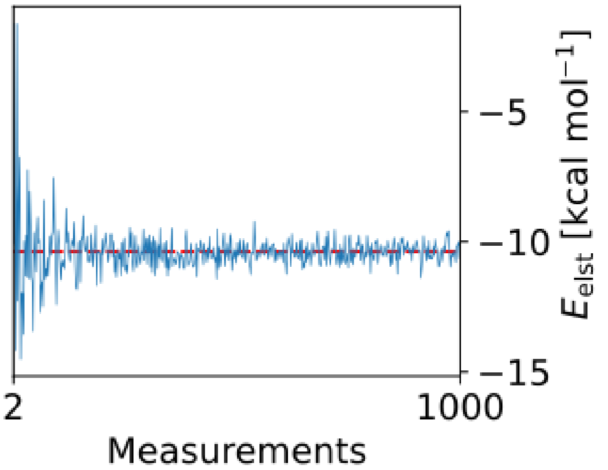

FIG. S8. The convergence of the electrostatics energy for intermediate A in dependence of the number of measurements, as in Fig. 4(c) in the main text.

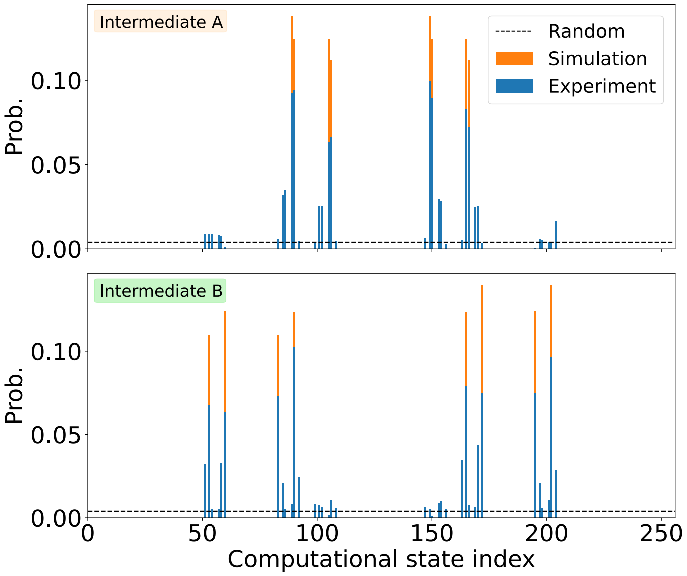

FIG. S9. The output statistics of the VQE circuit as in Fig. 4(a) after excluding all computational states with incorrect alpha and beta particle numbers from the measured data.

S7. ERROR MITIGATION USING ZERO-NOISE-EXTRAPOLATION

In order to improve the quality of the results obtained on NISQ machines, several error mitigation techniques have been developed. Typically, such methods utilize statistical procedures, that require a large number of implementations of quantum circuits, to improve the results.

Because of this additional cost, that directly implies larger runtime on the quantum hardware, we first estimate the effect of error mitigation using a noisy hardware simulator. The dominating error source in the *aqt_marmot* hardware is depolarizing noise. Therefore, we emulate the noise using a depolarizing noise channel with a fixed error rate per gate. As mentioned in the main text, we assume error rates of 1.5% and 0.3% for the two qubit MS gates and the single qubit local gates.

A suitable error mitigation method for this kind of errors is Zero-Noise-Extrapolation (ZNE). In ZNE we scale the noise by artificially increasing the circuit length. The length of the quantum circuit is thereby increased according to the noise scaling parameter λ. For $\lambda = 1$ we implement the original circuit that is not altered. In general, we can use an arbitrary set of scaling parameters. After the implementation of the circuits with variable lengths, e.g. $\lambda = [1, 2, 3]$, we extrapolate the behavior of the device for the zero noise case $\lambda = 0$. For the extrapolation we define a figure of merit, typically an expectation value, and a fitting function. Depending on the scaling of the expectation value with noise we choose a suitable fitting function. This can be a linear, a polynomial, an exponential or any other function that is able to resemble the noise scaling.

We implement ZNE using the open source python toolkit *mitiq* [12]. As the noise scaling function, we choose to use the unitary folding strategy `fold_gates_at_random`, that is described in detail in [12]. The scaled circuits resemble the same unitary as the original circuit, where the total number of gates $n_\lambda \approx n_0\lambda$ is roughly the number of gates of the original circuit n_0 multiplied by the scaling factor λ.

As a testbed, we use the 8-qubit circuit to estimate the electrostatic energy of the quantum circuit corresponding to intermediate A, that contains a gate count of $(R_\mathrm{Z}, 168), (R_\mathrm{X}, 111), (R_\mathrm{XX}, 63)$ in the original version. ZNE is performed using the noisy simulator with scaling parameters $\lambda = [1, 2, 3]$, where each of the three circuits is implemented a total of 60000 times (300 noisy simulations with 200 shots each). Since the R_XX gate is not supported in *mitiq*, we transpile the circuit into the gate set $R_\mathrm{Z}, R_\mathrm{X}, CX$ (a controlled-X or controlled-NOT gate). To prevent the removal of the additional gates in the scaled circuits, we use the `optimization_level=1` option in the Qiskit transpiler. Therefore the gate count is slightly different compared to the original circuit $(R_\mathrm{Z}, 139), (R_\mathrm{X}, 121), (R_\mathrm{XX}, 63)$ for $\lambda = 1$, $(R_\mathrm{Z}, 140), (R_\mathrm{X}, 239), (R_\mathrm{XX}, 122)$ for $\lambda = 2$ and $(R_\mathrm{Z}, 140), (R_\mathrm{X}, 372), (R_\mathrm{XX}, 189)$ for $\lambda = 3$.

To extrapolate the electrostatic interaction energy, which is calculated through the 1-PDM given in Eq. (12), we extrapolate the frequencies of the computational states, using the following fit function

$$f(\lambda) = a + b\exp(-c\lambda) \tag{S13}$$

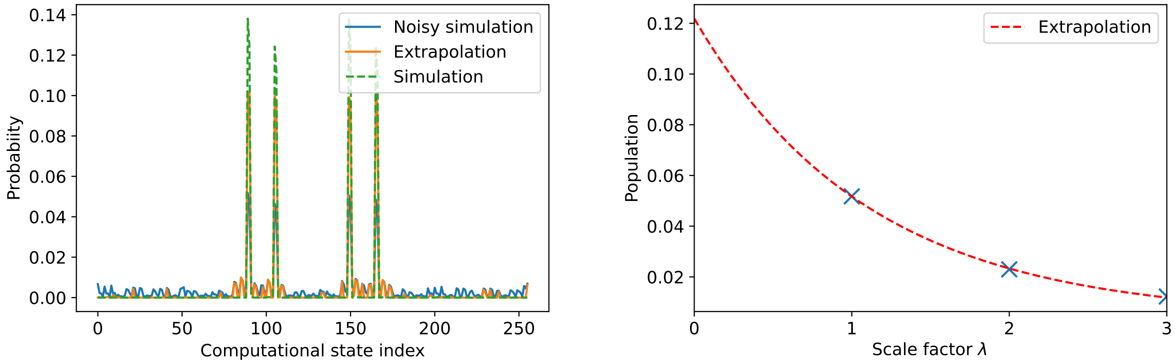

FIG. S10. (left) A comparison of the output of the noise-less and the noisy output statistics together with results obtained from extrapolating the frequencies to zero noise. (right) The output of the extrapolation of the computational state with highest frequency.

with an additional data point of $1/2^8$ for $\lambda = 10^5$, see Fig. S10(right) for the extrapolation of the computational state with highest frequency. All computational states with an extrapolated frequency < 0 are set to 0. The error of the extrapolated frequency is obtained by $\sigma^{(ZNE)}_{\text{freq.}} = \sqrt{\sigma_a^2 + \sigma_b^2}$, where σ_a and σ_b are the standard deviations of a and b obtained from the fit. In Fig. S10(left), we show a comparison between the noise-less, the noisy and the normalized extrapolated results. As visible, the extrapolation boosts the overlap w.r.t. the noise-less simulation. In fact, the Bhattacharyya distance of the noise-less simulation and the noisy simulation increases from 0.63 to 0.90 after extrapolation. We then utilize these extrapolated frequencies to reconstruct the 1-PDM, as per Eq. (12) in the main text, and subsequently compute the electrostatics. We propagate the errors from the extrapolation into the 1-PDM and into the electrostatics using standard Gaussian error propagation. Our findings indicate that the error in the electrostatics relative to the noise-free simulation did improve as anticipated from $\Delta E^{\text{noisy}}_{\text{elst.}} = 0.144$ kcal mol^{-1} to $\Delta E^{\text{ext.}}_{\text{elst.}} = 0.076 \pm 0.126$ kcal mol^{-1}. However, we note that the of the extrapolation is within the original value.

REFERENCES

[1] H. Shimizu, E. Obayashi, Y. Gomi, H. Arakawa, S.-Y. Park, H. Nakamura, S.-i. Adachi, H. Shoun, and Y. Shiro, Proton delivery in no reduction by fungal nitric-oxide reductase: Cryogenic crystallography, spectroscopy, and kinetics of ferric-no complexes of wild-type and mutant enzymes, Journal of Biological Chemistry **275**, 4816 (2000).

[2] R. Oshima, S. Fushinobu, F. Su, L. Zhang, N. Takaya, and H. Shoun, Structural evidence for direct hydride transfer from NADH to cytochrome P450nor, Journal of molecular biology **342**, 207 (2004).

[3] Molecular Operating Environment (MOE), 2022.02 Chemical Computing Group ULC, 910-1010 Sherbrooke St. W., Montreal, QC H3A 2R7, Canada, 2023. `https://www.chemcomp.com/Products.htm`, (accessed 2023-12-22).

[4] Promethium, `http://promethium.qcware.com`, QC Ware Corp, (accessed 2023-12-22).

[5] S. Sharma, A. A. Holmes, G. Jeanmairet, A. Alavi, and C. J. Umrigar, Semistochastic heat-bath configuration interaction method: Selected configuration interaction with semistochastic perturbation theory, Journal of chemical theory and computation **13**, 1595 (2017).

[6] A. A. Holmes, N. M. Tubman, and C. Umrigar, Heat-bath configuration interaction: An efficient selected configuration interaction algorithm inspired by heat-bath sampling, Journal of chemical theory and computation **12**, 3674 (2016).

[7] J. J. Goings, A. White, J. Lee, C. S. Tautermann, M. Degroote, C. Gidney, T. Shiozaki, R. Babbush, and N. C. Rubin, Reliably assessing the electronic structure of cytochrome P450 on today's classical computers and tomorrow's quantum computers, Proceedings of the National Academy of Sciences **119**, e2203533119 (2022).

[8] Q. Sun, T. C. Berkelbach, N. S. Blunt, G. H. Booth, S. Guo, Z. Li, J. Liu, J. D. McClain, E. R. Sayfutyarova, S. Sharma, et al., PySCF: the python-based simulations of chemistry framework, Wiley Interdisciplinary Reviews: Computational Molecular Science **8**, e1340 (2018).

[9] G.-L. R. Anselmetti, D. Wierichs, C. Gogolin, and R. M. Parrish, Local, expressive, quantum-number-preserving VQE ansätze for fermionic systems, New Journal of Physics **23**, 113010 (2021).

[10] H. R. Grimsley, S. E. Economou, E. Barnes, and N. J. Mayhall, An adaptive variational algorithm for exact molecular simulations on a quantum computer, Nature communications **10**, 3007 (2019).

[11] M. Loipersberger, F. D. Malone, A. R. Welden, R. M. Parrish, T. Fox, M. Degroote, E. Kyoseva, N. Moll, R. Santagati, and M. Streif, Accurate non-covalent interaction energies on noisy intermediate-scale quantum computers via second-order symmetry-adapted perturbation theory, Chemical Science **14**, 3587 (2023).

[12] R. LaRose, A. Mari, S. Kaiser, P. J. Karalekas, A. A. Alves, P. Czarnik, M. E. Mandouh, M. H. Gordon, Y. Hindy, A. Robertson, P. Thakre, M. Wahl, D. Samuel, R. Mistri, M. Tremblay, N. Gardner, N. T. Stemen, N. Shammah, and W. J. Zeng, Mitiq: A software package for error mitigation on noisy quantum computers, Quantum **6**, 774 (2022).
